# Avocado Consumption for 12 Weeks and Cardiometabolic Risk Factors: A Randomized Controlled Trial in Adults with Overweight or Obesity and Insulin Resistance

**DOI:** 10.1093/jn/nxac126

**Published:** 2022-06-14

**Authors:** Xuhuiqun Zhang, Di Xiao, Gabriela Guzman, Indika Edirisinghe, Britt Burton-Freeman

**Affiliations:** Department of Food Science and Nutrition, Center for Nutrition Research and Institute for Food Safety and Health, Illinois Institute of Technology, Chicago, IL, USA; Department of Food Science and Nutrition, Center for Nutrition Research and Institute for Food Safety and Health, Illinois Institute of Technology, Chicago, IL, USA; Department of Food Science and Nutrition, Center for Nutrition Research and Institute for Food Safety and Health, Illinois Institute of Technology, Chicago, IL, USA; Department of Food Science and Nutrition, Center for Nutrition Research and Institute for Food Safety and Health, Illinois Institute of Technology, Chicago, IL, USA; Department of Food Science and Nutrition, Center for Nutrition Research and Institute for Food Safety and Health, Illinois Institute of Technology, Chicago, IL, USA

**Keywords:** avocado, insulin resistance, Matsuda Insulin Sensitivity Index, cardiovascular risk factors, HbA1c

## Abstract

**Background:**

Diets emphasizing unsaturated fat and high fiber are associated with reducing cardiometabolic risk factors. Avocados are rich in MUFA and PUFA fats and fiber.

**Objectives:**

Assess replacement of carbohydrate energy with avocado energy for 12 wk on glucose homeostasis and cardiometabolic risk factors in self-selecting free-living adults who are overweight or with obesity and have insulin resistance.

**Methods:**

In a single-center, randomized, 2-arm, controlled, 12-wk parallel trial, adults [*n* = 93; male/female: 39/54; mean ± SD age: 42 ± 12 y; BMI: 32.6 ± 3.9 (in kg/m^2^);  HOMA-IR: 2.7 ± 1.7] were counseled to exchange avocado (AV) or control food (C; low fat, low fiber, energy matched) for carbohydrate food in their usual diet for 12 wk. The primary outcome was the change in Matsuda Insulin Sensitivity Index (MISI) after 12-wk interventions. Secondary outcomes were changes in fasting and post–oral glucose tolerance test glycemic variables, fasting lipids, endothelial activation and inflammation markers. Automated Self-Administered 24-h Dietary Assessment Tool captured weekly dietary intake. Intervention effects were mainly determined by ANCOVA using PC-SAS version 9.4.

**Results:**

Dietary total, MUFA, and PUFA fat; fiber; and vegetable intake were higher in the AV group compared with the C group (*P* < 0.05), and no change in body weight or composition was observed (*P* > 0.05). Differences between the changes in MISI after AV compared with C were not different (Δ0–12 wk, *P* = 0.1092). Differences in fasting insulin (Δ0–12 wk, *P* = 0.0855) and improved glycated hemoglobin (Δ0–12 wk, *P* = 0.0632) after AV compared with C were suggested. C-reactive protein was significantly lower after AV compared with C at 12 wk (*P* = 0.0418). Select biomarkers of endothelial activation and lipoproteins by NMR were also influenced by AV compared with C food intake.

**Conclusions:**

Avocado intake was associated with a healthier dietary pattern and trends favoring improved glucose control and reduced biomarkers of cardiometabolic risk when replacing avocado energy for carbohydrate energy in free-living adults who are overweight or with obesity and have insulin resistance. This trial was registered at clinicaltrials.gov as NCT 02695433.

## Introduction

Global estimates indicate 11 million deaths and 255 million disability-adjusted life years are attributable to poor diet (1). Higher intake of saturated fat, sodium, and refined carbohydrates and lower intake of fiber-rich foods (fruit, vegetables, and whole grains) is consistently associated with cardiometabolic risk factor traits, the incidence of metabolic syndrome, and death (1, 2). Suboptimal fruit and vegetable intakes are among the top 5 dietary risks contributing to cardiovascular diseases and type 2 diabetes mellitus (T2DM) (1). Insulin resistance (IR) is a central feature of cardiometabolic disease, affecting >30% of the US population (3). IR leads to impaired glucose tolerance, dyslipidemia, and endothelial dysfunction, playing an important role in the pathology and progression of T2DM, obesity, and hypertension and increasing the risk of cardiovascular diseases and all-cause mortality (4, 5).

Diets emphasizing unsaturated fat sources are associated with improved insulin sensitivity and glucose homeostasis (6–8). A meta-analysis of randomized controlled feeding trials in adults with normo- and hyperglycemia observed replacing carbohydrates with MUFA or PUFA sources improved glucose-insulin homeostasis measured by significantly lowering glycated hemoglobin (HbA1c) 0.09% and 0.11% unit%, respectively, and lowering HOMA-IR by 2.4% and 3.4%, respectively (6). Another meta-analysis of randomized controlled trials in patients with T2DM observed a high-MUFA diet significantly reduced fasting plasma glucose by 10.3 mg/dL compared with a high-carbohydrate diet (7). Insulin sensitivity was also reported to increase in individuals with prehypertension or stage 1 hypertension after a 6-wk unsaturated fat–rich diet compared with a carbohydrate-rich diet (8).

Avocado (*Persea americana*) is a nutrient-dense food with high MUFA, PUFA, dietary fiber, folate, potassium, and other essential nutrients and phytochemicals (9). Our previous research incorporating avocado in a breakfast test meal significantly reduced postprandial glucose and insulin concentrations, increased flow-mediated vasodilation, influenced postprandial lipoprotein metabolism (10), and increased satiety, including related satiety peptides (11), compared with an energy-matched high-carbohydrate breakfast. Wien et al. (12) reported reduced insulin concentrations after a test meal incorporating avocado energy but not when avocado was added to the meal. In a fully controlled feeding study, daily intake of avocado for 5 wk significantly decreased LDL particle number, small dense LDL cholesterol, and oxidized LDL from the baseline (13, 14). In a partially controlled feeding trial, Khan et al. (15) reported that daily intake of an avocado-containing meal for 12 wk had no significant effect on HOMA-IR and Matsuda Insulin Sensitivity Index (MISI) compared with intake of a control meal for 12 wk in adults who are overweight or with obesity (OW/OB).

Extending current knowledge, the present study aimed to investigate the effect of replacing carbohydrate energy with avocado energy for 12 wk in a free-living setting on cardiometabolic risk factors in adults with OW/OB and IR. We hypothesized that avocado intake would improve whole-body insulin sensitivity in individuals exhibiting IR as measured by the MISI, resulting in improved glucose control, lipids and lipoprotein variables, and markers of endothelial (dys)function and inflammation.

## Methods

### Ethics and participants

The Institutional Review Board of the Illinois Institute of Technology (IIT) reviewed and approved this study (IRB2016-001). The trial was registered at clinicaltrials.gov as NCT 02695433. All participants signed and dated the informed consent before any study-related procedures commenced. The clinical part of study was conducted from 2016 to 2021 (except the period halted for the COVID-19 pandemic lockdown) in the Center for Nutrition Research (CNR) at the IIT.

Adults with OW/OB and IR were recruited from the Chicago, Illinois, community and the surrounding region by newspapers, online advertisements, and local flyers. Participants were required to be in the age range between 25 and 65 y, BMI (in kg/m^2^) between 25 and 42 (inclusive), IR as defined by HOMA-IR ≥2, and abdominal OB as defined by midpoint waist circumference >102 cm for men and >88 cm for women. Individuals who smoked or used medications that would interfere with outcomes of the study (i.e., oral and injectable hypoglycemic medications, lipid-lowering medications, insulin-sensitizing medications), had allergies/intolerances to foods consumed in the study, consumed ≥3 avocados per week, consumed ≥3 servings of nuts per week, or had clinical evidence/history of diabetes or cardiovascular, respiratory, renal, gastrointestinal, or hepatic diseases that might interfere with study endpoints were not eligible to participate (**Supplemental Table 1**, inclusion and exclusion criteria).

### Study design

**FIGURE 1:**
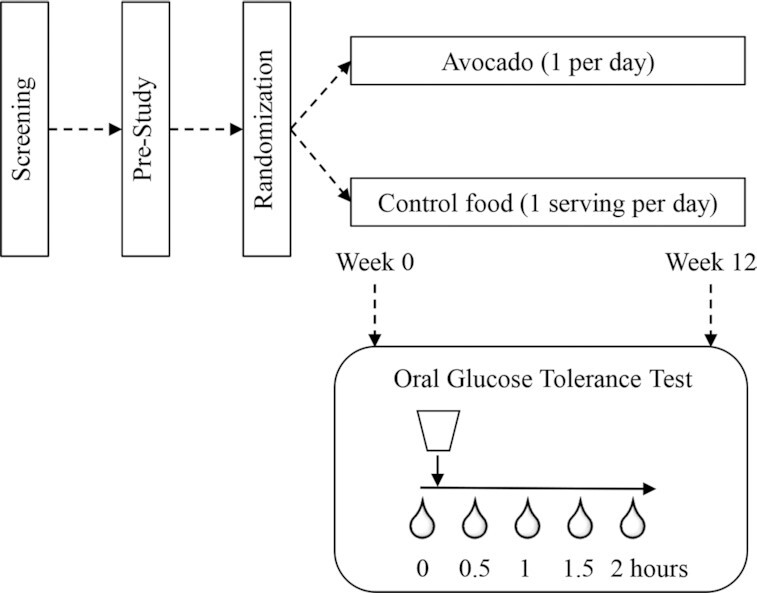
Study design schema.

This trial was a single-center, randomized, 2-arm, controlled, statistician-blinded, 12-wk parallel study. Adults were randomly allocated to consume an avocado or an energy-equivalent serving of control food daily for 12 wk (**Figure 1**). Adults who qualified were asked to maintain their usual level of physical activity and dietary habits with minor adaptions to accommodate the intervention. Food intake was monitored once per week using the Automated Self-Administered 24-h Dietary Assessment Tool (ASA24; version 2017). Study assessments were performed before and after 12-wk interventions, which included measuring anthropometric variables (height, weight, waist circumference, and body composition); blood pressure (BP); fasting plasma glucose and insulin concentrations; HbA1c; markers of endothelial activation [intercellular adhesion molecule 1 (ICAM-1) and vascular cell adhesion molecule 1 (VCAM-1)]; fasting lipid profile (total triglyceride, total cholesterol, HDL cholesterol, and LDL cholesterol]; lipoprotein particle size and concentrations, including subfractions; and inflammation biomarkers [high-sensitivity C-reactive protein (hsCRP), IL-6, and monocyte chemoattractant protein 1 (MCP-1)]. In addition, participants completed an oral glucose tolerance test (OGTT) for assessing postprandial glucose and insulin responses based on peak concentrations (C_max_), area under the glucose and insulin response curves (AUC), and calculating MISI. The primary outcome was change in insulin sensitivity as measured by MISI after 12-wk interventions. Secondary outcomes were changes in glycemic and lipid variables, endothelial activation, and inflammation markers. Tertiary outcomes were changes in lipoprotein variables by NMR, body weight, and composition.

### Intervention foods and dietary restriction

Participants were counseled to consume their usual diet with the exception of replacing study foods (1 Hass avocado or control low-fat/low-fiber/high-carbohydrate food) for a serving of carbohydrate-rich food they typically eat. Participants randomly allocated to the avocado arm were given weekly allotments of Hass avocados (size #48, ∼168 g pulp, provided by Hass Avocado Board) with varied recipe suggestions to consume 1 avocado/d, 7 d/wk (5–7 d was acceptable) for 12 wk. Participants randomly allocated to the control arm were given control foods in different combinations to match as closely as possible the energy level of 1 avocado per day. Foods such as mini bagels, pierogis, fruit juice, waffle, instant oatmeal, and others were provided (**Supplemental Table 2**). Control foods were purchased from PeaPod and local grocery stores and prepared/packaged by CNR staff weekly based on dispensing plan of randomization over the study period. Participants were advised to avoid all sources of avocado (unless provided by the study), nuts, squash, legumes, green tea, flaxseeds, and steel-cut oats throughout the study period. Participants were advised to avoid alcohol, tea, coffee, caffeinated beverages, berry products, and dark chocolate for 20 h before blood collection visits.

### Study procedures

Participants came to the CNR for 2 study day visits and 11 weekly pickup visits. The 2 study day visits occurred at week 0 (day 0; baseline) and the end of week 12 (day 84 ± 3). Participants arrived at the CNR fasted (10–12 h, confirmed by finger stick) and well hydrated on the morning of each scheduled study day visit. After assessing readiness based on protocol compliance (i.e., dietary restrictions and fasting), anthropometrics, body composition, and vital signs were measured. Body composition was measured with participants wearing a light robe provided by CNR and no shoes after emptying their bladder using the Tanita Body Composition Analyzer Model BC-418 (Tanita). Vital signs (BP and heart rate) were measured sitting, after a minimum resting period of 5 min, using an automatic sphygmomanometer (Omron digital blood pressure monitor, HEM-907XL). Systolic BP and diastolic BP values were an average of second and third values of 3 measurements separated by 5 min. An intravenous catheter was placed in the participants’ nondominant arm by a licensed health care professional. A fasting blood sample was collected (0 h), and participants were provided with an OGTT drink. Subsequent blood samples were collected at 0.5, 1, 1.5, and 2 h. After the 2-h blood collection, the catheter was removed, and participants were evaluated for safety before leaving the CNR.

### Blood analysis

Blood samples were collected in vacutainer tubes with EDTA coating, placed on ice, and centrifuged for 15 min (4°C, 453 × *g*) within 30 min of collection. Aliquots were stored at –80°C until analyzed. Plasma glucose, insulin, hsCRP, total triglyceride, total cholesterol, and HDL cholesterol were assessed using the Randox Daytona Automated Clinical Analyzer (Randox) with appropriate standards and quality controls. LDL cholesterol was calculated using Friedewald's equation. HbA1c was measured using DCA HbA1c reagent kits on DCA Vantage Analyzer (Siemens). ICAM-1 and VCAM-1 in plasma samples were measured using Quantikine ELISA assay methods (cat. SCD540 and SVC00, respectively; R&D Systems). IL-6 and MCP-1 in plasma samples were measured using high-sensitive ELISA assay methods (cat. HS 600B and DCP00, respectively; R&D Systems). All assay protocols were performed according to the manufacturers’ instructions, and appropriate quality controls were used as applicable. Intra- and interassay percent CVs were <10% in all the assays tested. Lipoprotein particles were analyzed using NMR spectra of frozen plasma specimens by LipoScience.

### Calculations and statistical analysis

Steady-state IR and β-cell function (i.e., HOMA-IR and HOMA-β) were calculated from fasting plasma glucose (FPG) and fasting plasma insulin (FPI) (16). AUCs for glucose and insulin during the OGTT were determined using the trapezoidal rule (17). MISI was calculated from FPG, FPI, mean glucose $\bar{\rm G}$, and mean insulin $\bar{\rm I}$ over 2-h OGTT (18), where glucose and insulin concentrations are expressed in mg/dL and μIU/mL, respectively. The equations are as follows:

(1) $$\begin{eqnarray*}
{\rm{HOMA\text{-}IR}} = \left( {{\rm{FPG}} \times {\rm{FPI}}} \right)/405
\end{eqnarray*}
$$

(2) $$\begin{eqnarray*}
{\rm{HOMA\text{-}\beta }} = \left( {360 \times {\rm{FPI}}} \right)/\left( {{\rm{FPG}}-63} \right)\%
\end{eqnarray*}
$$

(3) $$\begin{eqnarray*}
{\rm{MISI}} = 10,000/\sqrt {{\rm{FPG}} \times {\rm{FPI}} \times {\rm{\bar{G}}} \times {\rm{\bar{I}}}}
\end{eqnarray*}
$$

All statistical analyses were performed using PC-SAS (version 9.4; SAS Institute). Descriptive statistics and normality were performed on all outcome variables, and boxplot graphs were drawn to identify outliers. Outliers were identified in the HbA1c and hsCRP data sets and omitted in the final analysis with justification verified in study files (e.g., abnormal change, anemia, diarrhea, sprained leg/foot, weight gain). Participant demographic characteristics at baseline (week 0) were tabulated as mean ± SD or total number according to intervention randomization. Shapiro–Wilk tests (*P* > 0.05 was considered normal distribution) and Q–Q plots were used to assess the normality of raw data and residuals using UNIVARIATE and CAPABILITY procedures in SAS. Nonnormal variables, including insulin, IL-6, MCP-1, VCAM-1, lipoprotein variables, BMI, HOMA-IR, HOMA-β, and MISI, were log_10_ transformed and retested for normality before statistical analyses. ANCOVA was performed on the primary, secondary, and other outcome variables using the GLM procedure in SAS to test the main effects of the intervention (avocado compared with control) after 12 wk with baseline (week 0) as the covariate. Age, BMI, sex, and race were tested for significance in each model and included when significant and noted accordingly. Differences between the 12-wk changes in outcome variables after interventions were determined by subtracting each participant's baseline values from their postintervention values (weeks 0–12, delta Δ) and analyzed by ANCOVA with baseline values as the covariate. Cohen's *d* effect size and its CI were estimated as described previously (19). Cohen's *d* values of 0.2, 0.4, and 0.8 are considered small, medium, and large effect sizes, respectively, and provide insights about the magnitude of the difference between interventions (19, 20). Weekly ASA24 was averaged for months 1, 2, and 3 and analyzed by repeated-measures ANOVA for differences in dietary intake variables (kcals, carbohydrates, protein, etc.) between groups (avocado compared with control) over the 12-wk intervention period (effect of time) using the MIXED procedure in SAS. Mixed-model analysis of repeated measures using the MIXED procedure was also performed on time-course glucose and insulin concentrations to test the main effects of the intervention, week, and hour (0, 0.5, 1, 1.5, 2 h), including the respective 2-factor and 3-factor interactions with repeated effect of the participant. Restricted maximum likelihood with Kenward–Roger correction was used in MIXED models to minimize small sample size bias and to reduce bias due to missing values and the bias due to estimation of variance components (21). Outcomes data are shown as the unadjusted means and SEMs in tables and graphs unless otherwise stated. Statistical significance was based on a 2-sided comparison of intervention at the 5% significance level (*P* < 0.05) under a null hypothesis of no difference between treatments. Marginal statistical effects were acknowledged between the 5% and 10% significance level (*P* ≥ 0.05–0.10) in consultation with effect size estimates (20, 22).

Sample size estimates and randomization schedules were performed using SAS 9.4. Sample size was based on power calculations using PROC POWER and examining a range of expected responses and corresponding variance from previous studies (23, 24). A total evaluable data set of 96 OW/OB participants was sought, which required a sample size of 120 assuming randomization in a 1:1 ratio, 20–25% attrition, and mean difference of ∼0.42 units and SD of 0.7 for the primary endpoint (MISI). A blocked randomization schedule was produced using PROC PLAN for a 2-arm parallel design study. No stratification was included in the randomization. Participants were randomized to a code (blinded allocation) based on the blinded randomization schedule, and these codes were used on all documentation and labeling of tubes. Unblinded allocation assignments were sealed in an envelope until the end of the analyses. Participants were generally unblinded as they knew what they were eating. However, the study was referred to as a “diet plan,” and participants received interventions in dark bags so they did not know the foods other participants were receiving.

## Results

### Participant characteristics

**FIGURE 2:**
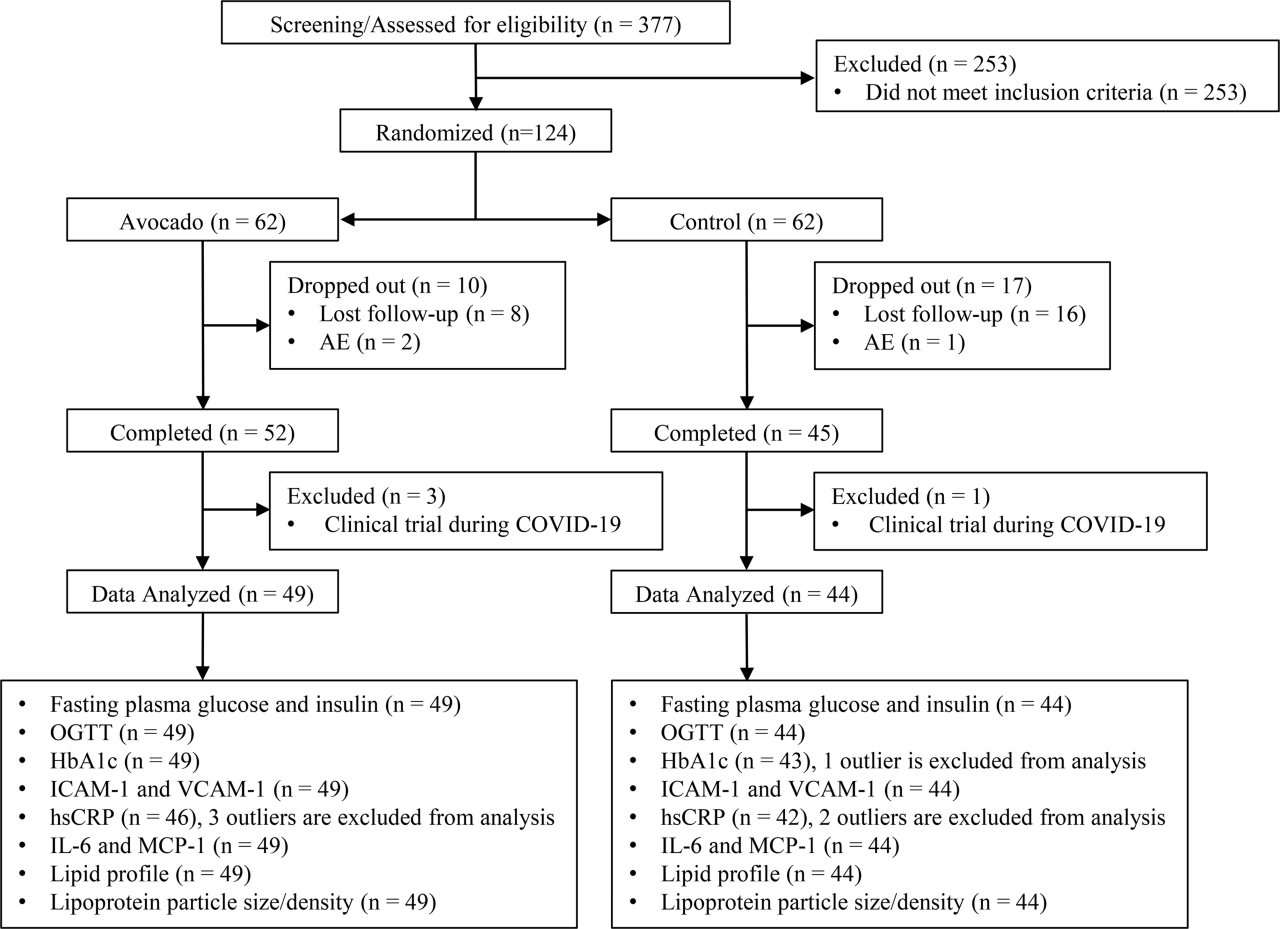
CONSORT flow diagram of the study. Adverse event (AE), ; COVID-19, coronavirus disease 2019; HbA1c, glycated hemoglobin; hsCRP, high-sensitivity C-reactive protein; ICAM-1, intercellular adhesion molecule 1; MCP-1, monocyte chemoattractant protein 1; OGTT, oral glucose tolerance test; VCAM-1, vascular cell adhesion molecule 1.

A total of 124 participants (avocado group, *n* = 62; control group, *n* = 62) were enrolled in the study (**Figure 2**). Ninety-seven participants (avocado group, *n* = 52; control group, *n* = 45) completed at least 12 wk of intervention; however, 4 participants (avocado group, *n* = 3; control group, *n* = 1) were in the study during the COVID-19 pandemic lockdown, in which case foods were mailed to participants. A final fasting blood sample was acquired, but with varied end of study visits outside the 12-wk intervention window, these 4 were excluded from data analysis. Ninety-three participants (*n* = 39 males, *n* = 54 females) were considered the evaluable data set for data analysis. Baseline characteristics are presented in **Table 1**.

**FIGURE 3:**
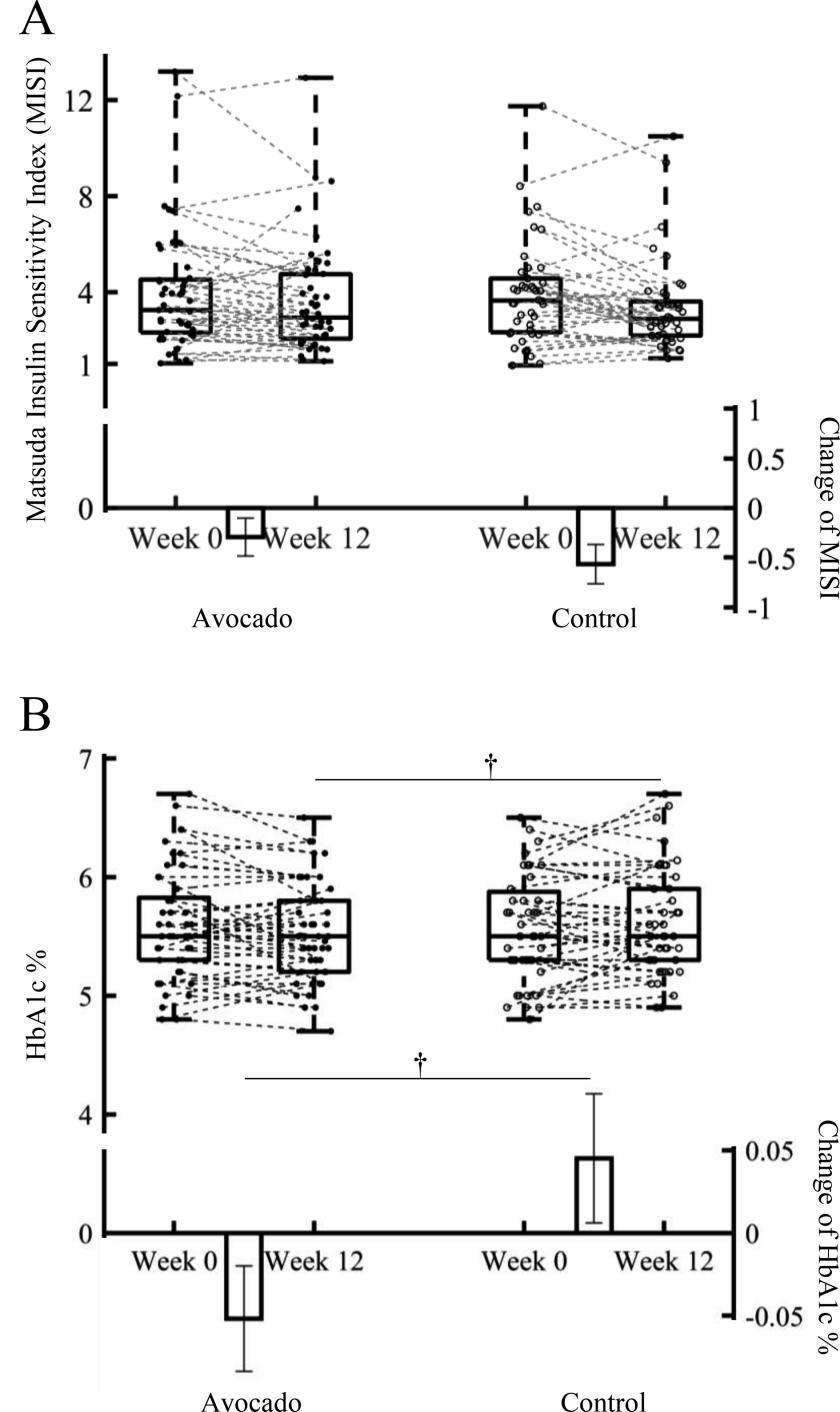
Box-and-whisker plots illustrate the distribution of the Matsuda Insulin Sensitivity Index (MISI) (A) and glycated hemoglobin (HbA1c) (B) values before and after 12-wk avocado or control intervention in adults who are overweight or with obesity and have insulin resistance. The line in the middle of the box is plotted at the median, and the inferior and superior limits of the box correspond to the 25th and the 75th percentiles, respectively. Bars represent mean change after 12-wk avocado or control food intervention with their standard errors; *n* = 49 and 44 for avocado and control groups, respectively. ANCOVA using the GLM procedure via PC-SAS was performed on the MISI and HbA1c to test the main effects of the intervention (avocado compared with control) after 12 wk with baseline (week 0) as the covariate. ^†^Marginal statistical effects were acknowledged at *P* = 0.05–0.1. GLM, general linear model.

**TABLE 1:** Baseline characteristics of study participants^1^

Variable	Avocado (*n* = 49)	Control (*n* = 44)
Age, y	40.6 ± 11.8	42.7 ± 12.5
Weight, kg	92.2 ± 14.7	95.6 ± 17.4
Waist,^2^ cm, midpoint	104 ± 10.5	106 ± 10.9
BMI, kg/m^2^	32.3 ± 3.90	32.8 ± 3.88
Fasting plasma glucose, mg/dL	106 ± 9.73	104 ± 11.1
Fasting plasma insulin, μIU/mL	10.3 ± 5.48	10.1 ± 5.67
HOMA-IR	2.72 ± 1.61	2.69 ± 1.70
Female/male, *n*	28/21	26/18
Race (Asian/His/Cau/AA/Other)	5/8/16/17/3	5/7/17/15/0

1 Data were obtained from week 0. Values are means ± SDs for continuous variables; counts are reported for categorical variables; *n* = 49 and 44 for avocado and control groups, respectively. AA, African American; Cau, Caucasian; His, Hispanic.

2 Waist circumference was measured at the midpoint between the lower margin of the least palpable rib and the top of the iliac crest.

### Dietary assessment

Significant intervention effects indicated the avocado group (*n* = 49) consumed significantly higher total fat, total MUFA and PUFA fat, dietary fiber, folate, and vegetables (avocado is categorized as a vegetable in ASA24) compared with the control group (*n* = 44) (*P* < 0.05, all; **Table 2**) consistently throughout the intervention. A significant time effect was observed in protein intake, indicating significantly increased protein intake in month 3 compared with month 2 (*P* = 0.0154) in all participants, independent of intervention allocation. The intervention by time interactions were not significant.

**TABLE 2:** Dietary assessment during 12-wk avocado or control intervention in adults who are overweight or with obesity and have insulin resistance^1^

	Avocado (*n* = 49)			Control (*n* = 44)			Intervention AV vs. C, *P* value^2^	Time *P* value^2^	Intervention × time *P* value^2^
Nutrient	Month 1	Month 2	Month 3	Month 1	Month 2	Month 3			
Energy, kcal/d	2130 ± 74.4	2080 ± 85.8	2200 ± 88.9	1900 ± 74.2	1890 ± 77.1	1990 ± 67.4	0.1259	0.3043	0.8386
Protein, g/d	85.4 ± 3.86	81.0 ± 3.76	91.2 ± 5.45	75.3 ± 3.81	73.8 ± 3.86	83.4 ± 4.17	0.1944	0.0209	0.8386
Total fat, g/d	100 ± 3.70	96.0 ± 4.03	103 ± 4.22	77.5 ± 4.13	71.5 ± 3.24	77.8 ± 3.09	0.0001	0.0780	0.9644
Total saturated, g/d	26.6 ± 1.28	27.7 ± 1.51	30.1 ± 1.85	25.8 ± 1.49	23.3 ± 1.35	25.8 ± 1.24	0.1150	0.1699	0.2195
Total monounsaturated, g/d	42.0 ± 1.55	40.8 ± 1.63	42.6 ± 1.71	27.6 ± 1.70	24.8 ± 1.14	27.1 ± 1.14	<0.0001	0.1578	0.8828
Total polyunsaturated, g/d	21.9 ± 1.11	19.6 ± 0.888	20.3 ± 0.95	17.5 ± 1.08	17.5 ± 0.873	18.2 ± 0.907	0.0301	0.2536	0.2164
Carbohydrate, g/d	220 ± 10.6	221 ± 10.9	224 ± 11.8	223 ± 7.93	232 ± 9.69	238 ± 9.74	0.6780	0.8370	0.5382
Sugar, g/d	83.0 ± 5.71	84.0 ± 6.31	88.1 ± 6.97	88.3 ± 4.40	97.2 ± 5.79	98.4 ± 6.94	0.4176	0.6520	0.5181
Fiber, total dietary, g/d	26.1 ± 1.03	24.9 ± 1.06	25.4 ± 1.08	14.8 ± 0.629	15.4 ± 0.606	15.9 ± 0.567	<0.0001	0.7317	0.4384
Calcium, mg/d	786 ± 48.5	810 ± 44.8	896 ± 52.2	897 ± 48.2	870 ± 50.1	1020 ± 60.2	0.3281	0.0071	0.6840
Potassium, mg/d	3090 ± 118	3020 ± 124	3270 ± 134	2350 ± 96.0	2420 ± 94.6	2600 ± 99.2	0.0007	0.0338	0.6792
Sodium, mg/d	3520 ± 152	3460 ± 176	3500 ± 155	3370 ± 134	3250 ± 133	3490 ± 119	0.5436	0.4007	0.7275
Vitamin C, mg/d	89.8 ± 9.11	88.9 ± 7.43	92.8 ± 6.94	72 ± 6.04	87.7 ± 6.51	94.4 ± 7.93	0.7143	0.2994	0.1653
Folate, total, μg/d	450 ± 18.3	462 ± 21.6	473 ± 21.6	360 ± 15.9	368 ± 16.5	398 ± 15.2	0.0031	0.1671	0.7002
Vitamin A, RAE, μg/d	578 ± 48.9	547 ± 41.9	636 ± 54.2	683 ± 44.0	691 ± 46.7	625 ± 35.2	0.3145	0.9714	0.1541
Cholesterol, mg/d	343 ± 23.9	324 ± 29.4	347 ± 26.1	301 ± 21.8	277 ± 19.2	303 ± 17.9	0.1949	0.4427	0.9685
Vitamin D (D2 + D3), μg/d	3.69 ± 0.442	4.11 ± 0.558	4.62 ± 0.513	4.38 ± 0.449	4.44 ± 0.519	4.96 ± 0.486	0.4946	0.3486	0.9370
Fruit,^3^ cup/d	0.745 ± 0.146	0.768 ± 0.117	0.717 ± 0.102	0.736 ± 0.0991	0.827 ± 0.0942	0.990 ± 0.0999	0.5726	0.8866	0.2498
Vegetables,^3,4^ cup/d	2.48 ± 0.138	2.39 ± 0.155	2.48 ± 0.137	1.60 ± 0.109	1.69 ± 0.122	1.64 ± 0.114	0.0001	0.8554	0.5225
Starchy vegetables,^3^ cup/d	0.291 ± 0.0433	0.305 ± 0.0454	0.34 ± 0.0445	0.388 ± 0.0541	0.419 ± 0.057	0.41 ± 0.053	0.1972	0.7687	0.8790
Grains, oz/d	5.63 ± 0.375	5.39 ± 0.33	5.43 ± 0.345	6.26 ± 0.335	5.87 ± 0.324	6.27 ± 0.260	0.3430	0.3228	0.8807
Whole grains, oz/d	0.788 ± 0.112	0.743 ± 0.115	0.859 ± 0.156	0.919 ± 0.137	0.733 ± 0.0875	0.899 ± 0.115	0.9916	0.3523	0.7065
Refined grains, oz/d	4.27 ± 0.355	4.25 ± 0.329	4.02 ± 0.297	4.64 ± 0.335	4.44 ± 0.341	4.80 ± 0.278	0.6295	0.6995	0.3614

1 Values are the unadjusted mean ± SEM of the raw data; *n* = 49 and 44 for the AV and C groups, respectively. Dietary assessment was conducted by weekly Automated Self-Administered 24-h Dietary Assessment Tool (ASA24). AV, avocado; C, control; Retinol Activity Equivalent (RAE),.

2 Compositional differences between the 2 dietary groups (AV compared with C) was assessed by comparing energy or nutrient intake variables (kcals, carbohydrates, protein, etc.) from individuals’ ASA24 over time (collected once per week for 4 wk) using the MIXED procedure in SAS to test the main effect of study interventions (AV compared with C), time (month 1 compared with month 2 compared with month 3) and their 2-factor interaction with repeated effect of the participant. Significant differences determined at a *P* value of <0.05. Bold values indicate significantly greater intake of nutrient/food in the avocado group compared with the control group independent of time (intervention effect, *P* < 0.05).

3 1 cup equivalent = 237 mL.

4 Avocado is classified as a vegetable in the ASA24 dietary analysis.

### Glycemic assessment

#### Insulin sensitivity by MISI

MISI was estimated from 2-h OGTT measurements of glucose and insulin concentrations (**Table 3** and **Figure 3**A). Mean MISI was not different between intervention groups at week 12 (*P* = 0.5430). The difference between mean changes in MISI (Δ0–12 wk) was also not different (Table 3, *P =* 0.1092), although a small to medium effect size of avocado intervention on MISI compared with the control was indicated (Cohen's *d* effect size: 0.305; 95% CI: –0.103, 0.715; **Supplemental Table 3**).

**TABLE 3:** Anthropometrics, blood pressure fasting metabolic indices, and oral glucose tolerance test indices before and after 12-wk avocado or control intervention, including mean changes (Δ), in adults who are overweight or with obesity and have insulin resistance^1^

	Avocado (*n* = 49)			Control (*n* = 44)			Week 12 AV vs. C, *P* value^2^	Δ0–12 wk AV vs. C, *P* value^2^
Variable	Week 0	Week 12	Δ0–12 wk	Week 0	Week 12	Δ0–12 wk		
BMI, kg/m^2^	32.2 ± 0.558	32.2 ± 0.623	–0.0449 ± 0.207	32.8 ± 0.592	32.9 ± 0.621	0.100 ± 0.127	0.5396	0.6238
Fat %	36.2 ± 1.14	36.2 ± 1.16	0 ± 0.273	37.3 ± 1.20	37.2 ± 1.26	–0.134 ± 0.219	0.6825	0.6825
Fat-free mass, kg	58.7 ± 1.79	58.9 ± 1.77	0.190 ± 0.248	59.7 ± 2.02	60.0 ± 2.10	0.323 ± 0.246	0.7251	0.7251
Systolic BP, mm Hg	124 ± 1.90	122 ± 1.86	–2.01 ± 1.24	126 ± 2.01	126 ± 2.41	0.0606 ± 1.86	0.2257	0.2257
Diastolic BP, mm Hg	77.5 ± 1.42	78.0 ± 1.52	0.497 ± 1.14	80.7 ± 1.75	80.5 ± 1.78	–0.250 ± 1.16	0.9884	0.9884
Fasting glycemic indices								
Fasting glucose, mg/dL	106 ± 1.40	107 ± 1.51	1.27 ± 1.07	104 ± 1.68	105 ± 1.51	0.788 ± 0.914	0.4770	0.4770
Fasting insulin, μIU/mL	10.2 ± 0.795	10.3 ± 0.727	0.0529 ± 0.533	10.1 ± 0.86	10.9 ± 0.677	0.774 ± 0.494	0.0951	0.0855
HOMA-IR	2.72 ± 0.233	2.76 ± 0.212	0.0358 ± 0.148	2.69 ± 0.259	2.83 ± 0.184	0.122 ± 0.141	0.1946	0.1098
HOMA-β	86.5 ± 6.05	85.9 ± 6.45	–0.547 ± 5.58	88.6 ± 5.91	110 ± 18.2	5.17 ± 3.46	0.0848	0.2106
HbA1c	5.59 ± 0.0654	5.54 ± 0.0589	–0.0518 ± 0.0326	5.58 ± 0.0702	5.60 ± 0.0671	0.0451 ± 0.0395	0.0574	0.0632
Oral glucose tolerance test								
Glucose AUC_0–2h_, mg/dL × h	292 ± 8.43	301 ± 8.42	9.33 ± 5.79	285 ± 9.33	290 ± 9.16	3.69 ± 5.88	0.3088	0.3723
Glucose C_max_, mg/dL	176 ± 6.09	180 ± 5.70	4.81 ± 3.97	171 ± 5.87	174 ± 5.70	1.50 ± 3.66	0.4012	0.3962
Insulin AUC_0–2h_, mg/dL × h	155 ± 12.5	163 ± 11.3	8.58 ± 6.59	158 ± 14.2	170 ± 13.2	11.5 ± 9.50	0.6981	0.7007
Insulin C_max_, mg/dL	125 ± 11.3	126 ± 9.42	1.08 ± 6.74	123 ± 10.8	133 ± 10.1	9.26 ± 7.86	0.3058	0.3835
MISI	3.91 ± 0.355	3.62 ± 0.324	–0.292 ± 0.191	3.95 ± 0.336	3.35 ± 0.292	–0.753 ± 0.254	0.5430	0.1092
Lipid profile								
Total triglyceride, mg/dL	114 ± 8.13	120 ± 10.9	6.22 ± 7.46	111 ± 8.88	107 ± 7.58	–4.07 ± 5.93	0.7333	0.2244
Total cholesterol, mg/dL	184 ± 5.11	182 ± 4.74	–1.61 ± 3.07	177 ± 5.03	181 ± 4.44	4.28 ± 2.97	0.3254	0.1943
HDL cholesterol, mg/dL	48.1 ± 2.15	48.4 ± 2.07	0.367 ± 1.03	45.4 ± 2.06	47.1 ± 2.25	1.64 ± 0.873	0.5321	0.2413
LDL cholesterol, mg/dL	113 ± 4.23	112 ± 3.92	–0.551 ± 2.67	110 ± 3.93	113 ± 3.94	3.32 ± 2.23	0.4406	0.3656
Inflammation biomarkers								
hsCRP, mg/L	3.96 ± 0.812	3.20 ± 0.577	–0.758 ± 0.673	3.55 ± 0.427	3.67 ± 0.498	0.120 ± 0.370	0.0418	0.1748
IL-6, pg/mL	3.15 ± 0.382	3.02 ± 0.373	–0.132 ± 0.203	3.77 ± 0.457	3.81 ± 0.493	0.0373 ± 0.208	0.5105	0.4410
MCP-1, pg/mL	210 ± 17.2	209 ± 19.0	–1.12 ± 7.07	193 ± 7.24	189 ± 6.33	–3.65 ± 4.73	0.5531	0.7466
Endothelial function biomarkers								
ICAM-1, ng/mL	208 ± 8.43	207 ± 9.10	–0.443 ± 3.85	213 ± 11.1	215 ± 10.7	1.23 ± 2.98	0.8742	0.6987
VCAM-1, ng/mL	629 ± 21.9	622 ± 15.6	–6.97 ± 15.0	674 ± 25.2	686 ± 23.1	11.1 ± 12.5	0.0849	0.0471

1 Values are the unadjusted mean ± SEM of the raw data; *n* = 49 and 44 for the AV and C groups, respectively. AV, avocado; BP, blood pressure; C, control; GLM, general linear model; HbA1c, glycated hemoglobin; hsCRP, high-sensitivity C-reactive protein; ICAM-1, intercellular adhesion molecule 1; MISI, Matsuda Insulin Sensitivity Index; MCP-1, monocyte chemoattractant protein 1; VCAM-1, vascular cell adhesion molecule 1.

2 ANCOVA was performed using the GLM procedure via PC-SAS version 9.4 and baseline (week 0) values as the covariate. Sex was also included in fasting insulin and HOMA-IR analyses as a significant covariate. Statistical significance was at *P* < 0.05. Marginal statistical effects were acknowledged at *P* = 0.05–0.1.

#### Glucose and insulin responses

**FIGURE 4:**
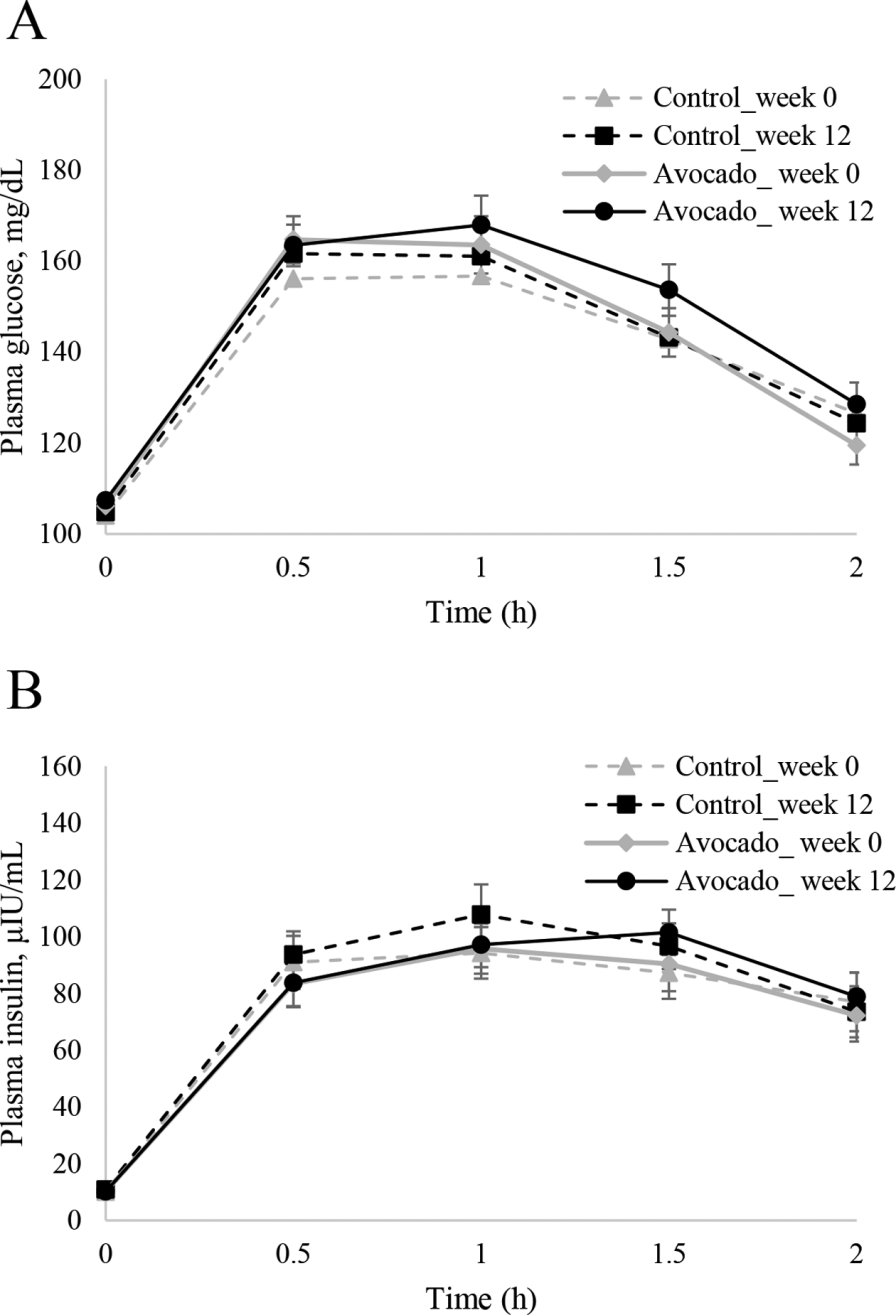
Plasma glucose (A) and insulin (B) responses over 2 h after the oral glucose tolerance test before and after the 12-wk avocado or control intervention in adults who are overweight or with obesity and have insulin resistance. Data are means ± SEMs; *n* = 49 and 44 for avocado and control groups, respectively. Mixed-model analysis of repeated measures using the MIXED procedure via PC-SAS was performed on glucose and insulin concentrations to test the main effects of the intervention (avocado compared with control), week (week 0 compared with week 12), and hour (0, 0.5, 1, 1.5, 2 h) and the respective 2-factor and 3-factor interactions (i.e., intervention × time, week × time, intervention × week, intervention × time × week) with repeated effect of the participant. No significant effects were observed.

Fasting glucose and insulin concentrations along with other glucoregulatory indices (i.e., HOMA-IR, HOMA-β) before and after 12-wk avocado and control interventions are shown in Table 3. Fasting insulin was marginally different between interventions at week 12 (*P =* 0.0951), as was the difference in the mean change response for fasting insulin (Δ0–12 wk, *P =* 0.0855) after controlling for sex (*P <* 0.01). HOMA-IR trended toward improvement with avocado intake but was not different from control (*P =* 0.1098, respectively). The effect size estimates for fasting insulin and HOMA-IR were relatively small (Cohen's *d* effect size: ≤0.2). No effect of the interventions was indicated for fasting glucose or glucose and insulin responses post-OGTT (glucose and insulin AUC_0–2h_,  glucose, and insulin C_max_; Table 3, **Figure 4**).

#### Plasma HbA1c

Avocado intake tended to lower HbA1c compared with control food intake after 12 wk. Differences in the means at 12 wk (5.54 ± 0.0589% compared with 5.60 ± 0.0671%, *P* = 0.0574) and the mean changes (Δ0–12 wk, *P =* 0.0632) are illustrated in Table 3 and Figure 3B. The pooled difference was –0.097% (95% CI: –0.198, 0.00402), which corresponded to a medium effect of the avocado intervention on HbA1c compared with control (Cohen's *d* effect size: –0.399; 95% CI: –0.810, 0.0122; Supplemental Table 3).

#### Anthropometrics, BP, proinflammatory cytokines, and markers of endothelial dysfunction

BMI, body composition (fat percent and fat-free mass), and BP did not change after the 12-wk interventions (*P* > 0.05, all; Table 3). Fasting plasma hsCRP was significantly lower after daily avocado intake compared with control food intake at 12 wk (3.20 ± 0.577 mg/L compared with 3.67 ± 0.498 mg/L, *P* = 0.0418, respectively, Table 3); however, the difference between mean changes was not different (*P* = 0.1748), and the effect size was relatively small (Cohen's *d* effect size: –0.238; 95% CI: –0.646, 0.171; Supplemental Table 3). Plasma IL-6, MCP-1, and ICAM-1 were not different between interventions at 12 wk, nor were mean changes different after the 12-wk interventions (Table 3). Differences in the change of VCAM-1 were evident (Δ0–12 wk, *P =* 0.0471, Table 3); however, the effect size estimate was small (Supplemental Table 3).

#### Lipids assessment

##### Plasma lipid profile

Total triglyceride, total cholesterol, and HDL and LDL cholesterol were not different between interventions at 12 wk, nor were the mean changes in response to interventions different (Table 3) (*P* > 0.05, all; Table 3).

##### Plasma lipoprotein particles by NMR

Lipoprotein particle subfractions were mostly unaffected by the interventions with the exception of modestly lower total LDL particle concentrations (968 ± 40.9 nmol/L compared with 1000 ± 40.0 nmol/L, respectively, *P* = 0.0694; **Table 4**) and lower small chylomicron/VLDL particle concentrations subfractions after daily avocado intake compared with control food intake at 12 wk (24.7 ± 1.72 nmol/L compared with 27.5 ± 1.50 nmol/L, respectively, *P* = 0.0638; Table 4). Differences in the change responses were also suggested (Δ0–12 wk, *P <* 0.09 for both). Effect size analysis supported a small to medium effect of avocado intervention on both total LDL and chylomicron/VLDL particle subfractions compared with the control (Cohen's *d* effect size: –0.316; 95% CI: –0.725, 0.0939 and –0.301; 95% CI: –0.710, 0.109, respectively; **Supplemental Table 4**).

**TABLE 4:** Lipoprotein variables by NMR before and after 12-wk avocado or control intervention in adults who are overweight or with obesity and have insulin resistance^1^

NMR analysis (variable)	Avocado (*n* = 49)			Control (*n* = 44)			Week 12 AV vs. C, *P* value^2^	Δ0–12 wk AV vs. C, *P* value^2^
	Week 0	Week 12	Δ0–12 wk	Week 0	Week 12	Δ0–12 wk		
Chylomicron/VLDL particle concentration, nmol/L								
Total	44.2 ± 2.73	40.7 ± 2.36	–3.90 ± 2.20	43.2 ± 2.13	42.3 ± 2.13	–0.928 ± 1.53	0.1732	0.3124
Large	4.25 ± 0.459	4.38 ± 0.464	0.148 ± 0.299	4.06 ± 0.429	3.75 ± 0.356	–0.307 ± 0.335	0.7244	0.2090
Medium	12.8 ± 1.23	11.7 ± 0.978	–1.16 ± 0.998	12.2 ± 1.28	11.0 ± 0.989	–1.17 ± 1.25	0.4203	0.9906
Small	27.2 ± 2.15	24.7 ± 1.72	–2.90 ± 1.86	26.9 ± 1.50	27.5 ± 1.50	0.553 ± 1.48	0.0638	0.0797
LDL particle concentration, nmol/L								
Total	977 ± 42.3	968 ± 40.9	–1.31 ± 19.0	960 ± 36.8	1000 ± 40.0	43.5 ± 23.3	0.0694	0.0897
IDL	250 ± 21.0	230 ± 16.9	–22.6 ± 18.0	257 ± 25.3	231 ± 17.1	–26.0 ± 20.3	0.9624	0.9498
LDL, large	108 ± 19.1	139 ± 21.4	30.1 ± 14.3	127 ± 22.2	156 ± 21.7	29.6 ± 23.7	0.7288	0.8088
LDL, small	619 ± 41.6	599 ± 40.0	–8.81 ± 25.9	577 ± 40.8	617 ± 41.1	39.8 ± 26.1	0.3468	0.1594
HDL particle concentration, nmol/L								
Total	32.6 ± 0.967	33.1 ± 0.943	0.408 ± 0.559	31.4 ± 0.698	31.6 ± 0.755	0.198 ± 0.459	0.8029	0.5320
Large	6.73 ± 0.497	6.93 ± 0.510	0.177 ± 0.228	6.57 ± 0.479	6.64 ± 0.525	0.0698 ± 0.204	0.6158	0.7061
Medium	10.9 ± 0.877	11.5 ± 0.876	0.442 ± 0.789	11.6 ± 0.749	11.9 ± 0.817	0.356 ± 0.650	0.4866	0.9114
Small	14.9 ± 0.867	14.6 ± 0.951	–0.210 ± 0.707	13.2 ± 0.886	13.0 ± 0.977	–0.242 ± 0.739	0.4147	0.6770
Average particle size, mm								
VLDL	51.9 ± 0.958	52.4 ± 1.02	0.627 ± 0.796	50.9 ± 0.930	50.8 ± 0.889	–0.177 ± 0.731	0.3726	0.2979
LDL	20.2 ± 0.075	20.3 ± 0.0887	0.154 ± 0.0752	20.2 ± 0.0826	20.3 ± 0.0892	0.0833 ± 0.0739	0.8590	0.6212
HDL	9.48 ± 0.0661	9.46 ± 0.0672	<–0.00 ± 0.0333	9.47 ± 0.0757	9.45 ± 0.0706	–0.0186 ± 0.0360	0.7563	0.7553

1 Values are the unadjusted mean ± SEM of the raw data; *n* = 49 and 44 for the AV and C groups, respectively. AV, avocado; C, control; IDL, intermediate-density lipoprotein; GLM, general linear model.

2 ANCOVA was performed using GLM procedure via PC-SAS version 9.4 and baseline values as the covariate. Age was also included in total LDL and small LDL analyses as a significant covariate. Statistical significance was at *P* < 0.05. Marginal statistical effects were acknowledged at *P* = 0.05–0.1.

## Discussion

The present study investigated replacing carbohydrate energy with avocado energy (∼1 avocado) daily for 12 wk on glucose homeostasis and cardiometabolic risk factors in free-living, self-selecting adults with OW/OB and IR. The primary endpoint was the change in insulin sensitivity evaluated by MISI. Results indicated that changes in MISI were not different between interventions. Alternatively, improved glucose control was suggested by modestly lower HbA1c after 12-wk avocado compared with the control food intervention, and this was supported by a medium effect size of the avocado intervention on HbA1c. Likewise, there was a trend for lower fasting insulin after 12-wk avocado compared with control food intake. With few exceptions, markers of vascular dysfunction, inflammation, lipids, and lipoprotein variables were not influenced by the dietary interventions. hsCRP and VCAM-1 were significantly reduced after the avocado intervention with small effect sizes, and total LDL and small chylomicron/VLDL particle concentrations trended lower after the avocado intervention compared with control and had a small to medium effect size. Eating avocados increased participants’ total fat intake, specifically MUFA and PUFA, and increased fiber, folate, and total vegetable intake compared with the control group. The data suggest that even with minimal intervention (an avocado a day for 12 wk), dietary and clinical changes can be observed to have potentially important implications on health status.

IR is a major risk factor in the development of prediabetes and T2DM. Methods to detect IR are available but have known limitations. The hyperinsulinemic-euglycemic clamp and the hyperglycemic clamp are recognized as the gold standards for measuring whole-body IR and β-cell function; however, the clamp methods are time-consuming, difficult to perform, and hardly applied in ordinary clinical practice (25, 26). HOMA uses basal-state (fasting) glucose and insulin (or C-peptide) concentrations to model tissue sensitivity to insulin and β-cell function (16). The HOMA-IR and HOMA-β correlate well with estimates using the hyperinsulinemic-euglycemic clamp, although they are limited in estimating suppression of hepatic glucose production and improvement of peripheral glucose uptake by postprandial insulin concentrations (25). The MISI, calculated from plasma glucose and insulin concentrations in the fasting state and during a 2-h OGTT, is a surrogate method for more accurately assessing liver and peripheral tissue insulin sensitivity (18). A clinical study of 153 participants, including individuals with normal glucose tolerance, impaired glucose tolerance, and T2DM, suggested the MISI highly correlated with the rate of insulin-mediated glucose disposal during the euglycemic insulin clamp (18). Suggested cutoff values for defining insulin sensitivity by the MISI are inconsistent across the literature, ranging from >2.5 to >6.4 as cutoffs for insulin sensitivity (27). In our previous research, individuals with prediabetes and IR had an MISI of 3.4 (95% CI: 2.8, 4.0), whereas metabolically healthy individuals had an MISI of 9.6 (95% CI: 6.8, 12.4) (28). In the current study, individuals had an MISI of 3.93 ± 0.24.

Including avocado in the diet regularly was hypothesized to improve insulin sensitivity in individuals with insulin resistance attributed to the bioactive components of avocado. Ahmed et al. (29) observed that avocado-derived lipid, avocatin B (Avo-B), a mixture of avocadyne and avocadene, improved glucose tolerance, glucose utilization, and insulin sensitivity (HOMA-IR) in a diet-induced obesity mouse model after 5 wk of supplementation. The authors suggested the results may be associated with Avo-B's fatty acid oxidation inhibitory effect (30). Avocados are also a rich source of unsaturated fat. Maki et al. (31) observed that the replacement of refined starches and added sugar with egg protein and unsaturated fats significantly increased peripheral insulin sensitivity from baseline, reporting an 18.1% increase in MISI after 3 wk of intervention, whereas the carbohydrate diet significantly decreased MISI from baseline by 5.7% in adults with IR (*n* = 25; baseline MISI: 1.34 ± 0.12). Collectively, these results warrant research of avocado intake on insulin sensitivity, particularly considering the combination of unsaturated fats and newly identified lipid molecules (Avo-B) having functional effects. Of consideration is that these avocado components may rely on population characteristics and/or dosing strategies to achieve dietary or pharmacokinetic targets that in turn manifest a biological outcome. The present study did not show significantly different MISI group means between control and avocado intervention at 12 wk, as was observed by Maki et al. (31), after a shorter 3-wk intervention. Maki et al.’s study population had a baseline MISI of 1.34 ± 0.12, whereas the baseline MISI of our study group was 3.93 ± 0.24, suggesting an unsaturated fat–rich diet may be more effective in individuals with poorer insulin sensitivity. Alternatively, higher intake concentrations of unsaturated fat and/or Avo-B may be required to increase insulin sensitivity (significantly) when it is less impaired. In accordance with our results, HOMA-IR and MISI were not different between avocado and control interventions after 12 wk in adults with OW/OB and who had less impaired insulin sensitivity (*n* = 105; baseline MISI: 5.7) (15).

HbA1c is a biomarker of glucose homeostasis representing average glycemic control over the past 2–3 months and accounts for both preprandial and postprandial blood glucose concentrations (32). Reduced HbA1c has been observed in streptozotocin-induced diabetic rats after an 8-wk intervention with different avocado extract-solvent fractions (n-hexane, chloroform, ethyl acetate, and n-butanol) (33). Data from the current study indicated a difference between the responses to interventions (–0.097%; –0.198%, 0.00402%; Supplemental Table 3) comparable to findings of meta-analyses of isocaloric replacement studies on glucose-insulin homeostasis (6, 7). Isocaloric replacement of 5% dietary energy from either carbohydrate or saturated fat with 5% dietary energy from either MUFA or PUFA significantly lowered HbA1c by 0.09–0.15% unit, independent of affecting glucose concentrations (6). In our study, we aimed to replace between 5% and 10% of carbohydrate energy with avocado energy or control carbohydrate food in free-living participants. Our dietary monitoring data suggest individuals were more likely to add the avocado to their diet compared with replacing carbohydrate calories because individuals in the avocado arm consumed ∼1 more vegetable serving than the control group (avocado counts as a vegetable in ASA24) and corresponding nutrients (fiber, potassium, folate, total fat, and monounsaturated fat) increased, whereas carbohydrate intake was relatively stable (Table 2). Our findings suggest a favorable effect of avocado on glucose homeostasis and support further research to identify the mechanisms of avocado-associated glycemic actions and whether strictly lowering carbohydrates while increasing avocado intake will amplify the effect size.

Previous work indicated lower concentrations of triglyceride-rich lipoproteins and higher concentrations of larger HDL particles after an acute meal challenge with avocado compared with isocaloric control meal in adults with OW/OB (10). These results were not replicated in this 12-wk avocado compared with control intervention. Only a trend for decreased small VLDL particle concentration and total LDL particle concentration was observed (Table 4) after avocado compared with control diet. One potential interpretation is the saturated fat content from the background diet remained relatively similar and high for both groups (avocado compared with control: 28 ± 2 g compared with 25 ± 1 g, respectively; Table 2), which may attenuate the effect of avocado and the higher MUFA intake. Dietary Guidelines for Americans recommend limiting calories from saturated fats to <10% of the total calories consumed each day, that is, ∼ 20 g/2000-calorie diet (34). High saturated fat (18% saturated fat) intake has been associated with increased concentrations of the small, medium, and large LDL particles (35, 36). Accordingly, replacing saturated fat with MUFA from avocado in a fully controlled 5-wk feeding study reduced fasting small, dense LDL cholesterol and increased the average LDL particle size in OW/OB adults (13).

Other emerging risk factors for cardiometabolic risk include endothelial function and inflammatory markers. Previous work with avocados in a weight-loss study revealed trends for decreased serum IL-1β and hsCRP beyond that of the weight loss (37). Plasma/serum hsCRP is elevated in chronic conditions such as obesity, cardiovascular disease (CVD), and diabetes (38). The role of hsCRP in disease, particularly atherosclerotic CVD disease, has attracted much attention over the past several decades, exploring it as a predictor and marker of disease but also as a target of interventions in the treatment and lowering of disease risk. Participants in our study, on average, started with hsCRP concentrations that classify them in the high-risk CVD range (39). We observed decreased hsCRP after avocado intake that was significantly lower than after the control intervention. Dietary fatty acid profiles with increased PUFA and MUFA have been associated with lower hsCRP (40, 41), which may provide insights into the current findings. PUFA and MUFA intakes were significantly higher in the avocado group compared with the control group over the course of the study. This dietary shift may have changed plasma fatty acid profiles influencing inflammatory pathways. Various data sets from animal and human studies corroborate the link between fat (amount and type) and inflammation (42), and Henning et al. (37) reported shifts in plasma fatty acid profiles consistent with their 12-wk avocado intervention.

The study has strengths and limitations. This was a randomized-controlled, 12-wk, parallel-designed free-living clinical research study that allowed for understanding the effects of a modest and relatively simplistic dietary intervention on cardiometabolic risk factors in a diverse group of adults. Free-living self-selecting conditions provide insight into how food will be incorporated and compensated for in the diet; however, this freedom may have been a limitation in the current study. Participants did not fully replace carbohydrate energy with avocado energy, which may have attenuated the effect size for certain outcome variables. Nonetheless, the addition of avocado to the diet revealed benefits and did not increase body weight. The dropout rate was 21.8%, which is comparable to other 3-mo intervention studies but may be considered a limitation with a per-protocol compared with intent-to-treat analysis. Testing of multiple secondary outcome variables may increase risk of making a type I error. A strength of this research is the race/ethnic diversity of the study population. An equal number of Caucasian and black/African American individuals participated in this study, comprising two-thirds of the sample size, and the remaining one-third self-reported Asian, Hispanic, and other affiliations. Race/ethnicity was included in models to account for variance when appropriate; future research should continue to enroll diverse populations.

In conclusion, avocado intake for 12 wk showed beneficial effects on glucose control suggested by the HbA1c results in adults with OW/OB and insulin resistance. Other insights included effects on fasting insulin, systemic and vascular inflammation markers, and modest effects on lipoprotein particle variables worth following up. Incorporating fresh avocados in the diet regularly can also help people achieve dietary recommendations to eat more fruit/vegetables and increase nutrients of concern, including fiber, potassium, and folate.

## Supplementary Material

nxac126_Supplemental_File

## Notes

Supported by Hass Avocado Board, Mission Viejo, CA, USA.

Author disclosures: The authors report no conflicts of interest.

Supplemental Tables 1–4 are available from the “Supplementary data” link in the online posting of the article and from the same link in the online table of contents at https://academic.oup.com/jn/.

Abbreviations used: ASA24, Automated Self-Administered 24-h Dietary Assessment Tool; Avo-B, avocatin B; BP, blood pressure; CNR, Center for Nutrition Research; CVD, cardiovascular disease; FPG, fasting plasma glucose; FPI; fasting plasma insulin; HbA1c, glycated hemoglobin; hsCRP, high-sensitivity C-reactive protein; ICAM-1, intercellular adhesion molecule 1; IIT, Illinois Institute of Technology; IR, insulin resistance; MCP-1, monocyte chemoattractant protein 1; MISI, Matsuda Insulin Sensitivity Index; OB, obesity; OGTT, oral glucose tolerance test; OW, overweight; T2DM, type 2 diabetes mellitus; VCAM-1, vascular cell adhesion molecule 1.
