# Supplementary material for: Avocado Consumption for 12 Weeks and Cardiometabolic Risk Factors: A Randomized Controlled Trial in Adults with Overweight or Obesity and Insulin Resistance
Source: J Nutr. 2022 Jun 14;152(8):1851–61. doi: 10.1093/jn/nxac126 (PMC9486596; doi:10.1093/jn/nxac126)
Supplement: nxac126_Supplemental_File [file nxac126_supplemental_file.docx]

Avocado consumption for 12 weeks and cardio-metabolic risk factors: a randomized controlled trial in adults with overweight or obesity and insulin resistance, Xuhuiqun Zhang et al. “Online Supplementary Material”

**Supplementary Data**

Supplementary Table 1. Inclusion and exclusion criteria.

| Inclusionary (Must meet all to participate) | Exclusionary (not qualified if meet *any)* |
| --- | --- |
| - Non-smoking^1^ man or woman - BMI between 25 and 42 kg/m^2^ - Aged 25-65 years old - HOMA-IR^2^ greater than or equal to 2.0 - Abdominal Obesity: mid-point waist circumference >102 cm for men or >88 cm for women - No clinical evidence/history of diabetes, cardiovascular, respiratory, renal, gastrointestinal, or hepatic diseases that may interfere with study endpoints as determined by study investigators - Able to provide informed consent and comply with study procedures | - Smoker - Vegan - Taking over the counter medication and/or supplements^3^ (e.g. fiber or protein supplements, probiotics and/or prebiotics, antioxidants, anti-inflammation, energy drinks) - Taking prescription medications that may interfere with study procedures or endpoint (e.g. oral and injectable hypoglycemic medications, gastrointestinal medications, antibiotics, diuretics, lipid lowering medications, insulin sensitizing medications) within the last 30 days - Working overnight (e.g. 3^rd^ shift of overnight workers) - Have cancer other than non-melanoma skin cancer in previous 5 years that may interfere with study endpoints as determined by study investigators - Taking unstable dose of hormonal contraceptive and/or stable dose less than 6 months - Planning to become pregnant, pregnant and/or breast-feeding - Excessive exercisers or trained athletes - Excessive coffee/tea drinker - Donated blood within last 3 months - Have allergies/intolerances to foods consumed in the study - Actively losing weight/ trying to lose weight (unstable body weight fluctuations of > 5 kg in 3 months) - Addicted to drugs and/or alcohol - Have significant psychiatric or neurological disturbances. - Excessive tree nut consumer^3^ - Consuming 3 or more avocados per week |

^1^ Past smokers may be allowed in the study if stopped > 2 years.

^2^ HOMA-IR (Homeostatic Model Assessment of Insulin Resistance) is calculated from fasting glucose and insulin values.

^3^ Nut consumers and those taking dietary supplements that may interfere with study results may be allowed in the study if they agree to stop consumption 30 days before start of study and limit consumption of those products for the duration of the 12-week study.

Supplementary Table 2. Nutrients contents of avocado and control foods per serving.^1^

| **Item Name** | **Wgt (g)** | **Cals (kcal)** | **Prot (g)** | **Fat (g)** | **SatFat (g)** | **MonoFat (g)** | **PolyFat (g)** | **Carb (g)** | **Sugar (g)** | **Fiber (g)** |
| --- | --- | --- | --- | --- | --- | --- | --- | --- | --- | --- |
| Avocado, HASS, Whole | 168 | 280 | 3 | 26 | 3 | 17 | 3 | 15 | 0 | 11 |
| bagel, plain, mini | 86 | 240 | 8 | 2 | 0 | 0 | 0 | 48 | 6 | 2 |
| Potato and Cheddar Pierogies | 152 | 227 | 7 | 3 | 1 | - | - | 44 | 1 | 1 |
| Strawberry Banana Fruit Smoothie Blend 4 Juice, Odwalla | 431 | 220 | 2 | 0 | - | - | - | 55 | 44 | 2 |
| Waffles, Nutri Grain, frozen, lowfat | 140 | 281 | 9 | 5 | 1 | 1 | 2 | 55 | 5 | 6 |
| Quaker Instant Oatmeal Fruit & Cream Variety, Strawberries & Cream | 70 | 260 | 6 | 4 | 1 | 2 | 1 | 54 | 24 | 4 |
| Mango Slices, Dried, Peapod | 80 | 240 | 4 | 0 | 0 | - | - | 64 | 60 | 4 |
| Peapod Corn Flakes Cereal | 70 | 250 | 5 | 0 | 0 | - | - | 60 | 5 | 3 |
| Pad Thai noodle soup, Vegan | 56 | 200 | 4 | 0 | 0 | - | - | 44 | 6 | 2 |
| Hash Browns, Simply Potatoes | 270 | 242 | 3 | 0 | 0 | - | - | 55 | 0 | 7 |
| Stone Bake French Dinner Roll 8ct, Pepperidge Farm | 100 | 240 | 8 | - | - | - | - | 50 | - | 2 |
| Rice Snacks, Chocolate Mini Peapod | 60 | 240 | 4 | 0 | 0 | - | - | 56 | 16 | 0 |
| Pudding, vanilla, snack cup JELL-O | 226 | 226 | 2 | 3 | 2 | - | - | 47 | 35 | 0 |
| Potato Gnocchi, Peapod | 112 | 270 | 8 | 1 | 0 | - | - | 57 | 2 | 2 |
| Peapod Graham Crackers Honey Low Fat | 70 | 280 | 4 | 4 | 0 | 0 | 2 | 56 | 16 | 4 |
| bagel, plain, mini | 86 | 240 | 8 | 2 | 0 | 0 | 0 | 48 | 6 | 2 |

^1^ Wgt, weight; Cals, calories; Prot, protein; SatFat, saturated fat; MonoFat, monounsaturated fat; PolyFat, polyunsaturated fat; Carb, carbohydrate.

Supplementary Table 3. Effect size analysis (Cohen’s d and 95% Confidence Interval) on anthropometrics, blood pressure, fasting metabolic indices and oral glucose tolerance test indices after 12-week avocado or control intervention in adults with overweight or obesity and insulin resistance^1^

| **Variables** | | **Cohen's d [95% CI]^1^** |
| --- | --- | --- |
| ΔBMI (kg/m^2^) | | -0.121 [-0.528, 0.287] |
| ΔFat% | | 0.0785 [-0.329, 0.486] |
| ΔFat-free mass (kg) | | -0.0788 [-0.486, 0.328] |
| ΔSystolic BP (mm Hg) | | -0.197 [-0.605, 0.212] |
| ΔDiastolic BP (mm Hg) | | 0.0951 [-0.312, 0.502] |
| **Fasting glycemic indices** | |  |
| ΔFasting glucose (mg/dL) | | 0.0710 [-0.336, 0.478] |
| ΔFasting insulin (μIU/mL) | | -0.204 [-0.613, 0.204] |
| ΔHOMA-IR^3^ | | -0.0871 [-0.494, 0.320] |
| ΔHOMA-β^3^ | | -0.176 [-0.584, 0.232] |
| ΔHbA1c^3^ | | -0.399 [-0.810, 0.0122] |
| **Oral glucose tolerance test** | |  |
| ΔGlucose AUC_0-2h_ (mg/dL×h)^3^ | | 0.143 [-0.265, 0.550] |
| ΔGlucose C_max_ (mg/dL) | | 0.127 [-0.280, 0.535] |
| ΔInsulin AUC_0-2h_ (mg/dL×h) | | -0.0544 [-0.462, 0.353] |
| ΔInsulin C_max_ (mg/dL) | | -0.167 [-0.575, 0.241] |
| ΔMatsuda Insulin Sensitivity Index | | 0.305 [-0.104, 0.715] |
| **Inflammation biomarkers** | |  |
| ΔIL-6 (pg/mL)^3^ | | -0.121 [-0.529, 0.286] |
| ΔMCP-1 (pg/mL)^3^ | | 0.0605 [-0.347, 0.468] |
| **Endothelial function biomarkers** |  |  |
| ΔICAM-1 (ng/mL)^3^ | | -0.0702 [-0.477, 0.337] |
| ΔVCAM-1 (ng/mL)^3^ | | -0.190 [-0.598, 0.218] |

^1^ Values are Cohen’s d effect size and 95% confidence interval (CI) estimated from changes of variables over 12 weeks in the avocado and control groups as previously described (1). The Cohen’s d values of 0.2, 0.4, and 0.8 were considered as small, medium, and large effect sizes, respectively (1).

Abbreviation: AUC, area under the curve; A BMI, body mass index; BP, blood pressure; HbA1c, hemoglobin A1c; HOMA-β, homeostatic model assessment for beta-cell function; HOMA-IR, homeostatic model assessment for insulin resistance; ICAM-1, intercellular adhesion molecule-1; IL-6, interleukin-6; MCP-1, monocyte chemoattractant protein-1; VCAM-1, vascular cell adhesion molecule-1.

Supplementary Table 4. Effect size analysis (Cohen’s d and 95% Confidence Interval) on lipoprotein variables by nuclear magnetic resonance (NMR) after 12-week avocado or control intervention in adults with overweight or obesity and insulin resistance .^1^

| **NMR Analysis (Variable)** | | **Cohen's d [95% CI]^1^** |
| --- | --- | --- |
| ΔChylomicron/VLDL Particle concentration (nmol/L) | Total | -0.228 [-0.636, 0.180] |
|  | Large | 0.213 [-0.195, 0.621] |
|  | Medium | 0.00185 [-0.405, 0.409] |
|  | Small | -0.301 [-0.710, 0.109] |
| ΔLDL Particle concentration (nmol/L) | Total | -0.316 [-0.725, 0.0939] |
|  | IDL^3^ | 0.0259 [-0.381, 0.433] |
|  | LDL, Large | 0.00439 [-0.403, 0.411] |
|  | LDL, Small | -0.277 [-0.686, 0.132] |
| ΔHDL Particle concentration (nmol/L) | Total | 0.0603 [-0.347, 0.467] |
|  | Large | 0.0731 [-0.334, 0.480] |
|  | Medium | 0.0174 [-0.39, 0.424] |
|  | Small | 0.00644 [-0.401, 0.414] |
| ΔAverage Particle size (mm) | VLDL | 0.155 [-0.253, 0.563] |
|  | LDL | 0.143 [-0.264, 0.551] |
|  | HDL | 0.0617 [-0.345, 0.469] |

^1^ Values are Cohen’s d effect size and 95% confidence interval (CI) estimated from changes of variables over 12 weeks in the avocado and control groups as previously described (1). The Cohen’s d values of 0.2, 0.4 and 0.8 were considered as small, medium, and large effect size, respectively (1).

Abbreviation: HDL, high-density lipoprotein; IDL, intermediate-density lipoprotein; LDL, low-density lipoprotein; VLDL indicated very-low-density lipoprotein.

Supplementary Data Reference:

1. Lee DK. Alternatives to P value: confidence interval and effect size. Korean J Anesthesiol [Internet]. 2016;69:555. Available from: http://ekja.org/journal/view.php?doi=10.4097/kjae.2016.69.6.555
